# Resistant Starch in MASLD: Microbial Ecology, Response Heterogeneity and the Path to Precision Nutrition

**DOI:** 10.3390/nu18203394

**Published:** 2026-10-16

**Authors:** Zihao Xu, Yifan Liu, Huikun Wu, Hai Xu, Li Zheng

**Affiliations:** The First Clinical College, Hubei University of Chinese Medicine, Wuhan 430061, China; 2527012087@stmail.hbucm.edu.cn (Z.X.); 2232701111@stmail.hbucm.edu.cn (Y.L.); 3398@hbucm.edu.cn (H.W.); 2016@hbucm.edu.cn (H.X.)

**Keywords:** resistant starch, MASLD, gut microbiome, precision nutrition, dietary fiber, microbial cross-feeding, treatment–effect heterogeneity, clinical translation

## Abstract

Resistant starch (RS) links a modifiable dietary exposure to microbial metabolism and hepatic health in metabolic dysfunction-associated steatotic liver disease (MASLD). A four-month randomized trial of 40 g/day high-amylose maize RS type 2 (HAM-RS2) reduced liver fat, while linked cohort analyses identified substantial variation in response. International trials in other metabolic populations reported null hepatic-fat effects, bringing population and nutritional context into focus. This critical narrative review examines the ecological basis of response variation and its implications for precision nutrition. Three interacting determinants organize the evidence: the preparation consumed, the functional capacity of the recipient microbiota and the host environment in which fermentation occurs. Substrate accessibility, primary degradation and microbial cross-feeding shape the metabolites available to the host. Disease-specific transfer and perturbation experiments support microbial amino-acid and inflammatory pathways; short-chain fatty acid (SCFA) metabolism, bile-acid handling and lipid absorption provide additional candidate links to hepatic response. Quantitative comparisons place RS alongside effective dietary and lifestyle interventions. We connect this framework to a concrete HAM-RS2 research regimen and a staged path from response prediction to comparative treatment benefit. Independent replication, reproducible functional measurements and marker-stratified trials can establish whether measuring microbial ecology improves nutritional choices alongside standard MASLD care.

## 1. Introduction

Metabolic dysfunction-associated steatotic liver disease (MASLD) places nutritional management at the intersection of liver disease and cardiometabolic health. The contemporary definition requires hepatic steatosis together with cardiometabolic risk, while distinguishing other causes and patterns of steatotic liver disease [1]. Dietary quality, physical activity, sustained weight management and treatment of metabolic comorbidities remain central to care [2]. Nutritional management includes reducing excess energy intake when appropriate and replacing saturated with unsaturated fats within a high-quality dietary pattern. Phase 3 trials of resmetirom and semaglutide have demonstrated histological benefits in selected patients with steatohepatitis and fibrosis [3,4]. Within this therapeutic setting, nutritional adjuncts should be evaluated for their contribution to specific hepatic and metabolic outcomes.

The gut microbiome offers a route through which diet can influence hepatic metabolism. Microbial processing changes the compounds presented to the intestinal epithelium and liver, while barrier function and immune signaling connect luminal activity with systemic physiology [5]. Resistant starch (RS), the starch fraction that escapes small-intestinal digestion, is a starch-based component of dietary fiber available for colonic fermentation [6]. Its structural diversity and measurable microbial degradation make it useful for investigating diet–microbiome relationships. Reduced small-intestinal carbohydrate availability can affect postprandial glycemia; colonic fermentation adds a distinct route to host metabolism. A prebiotic designation requires selective microbial utilization linked to a host benefit [7], which should be evaluated for the specified preparation and clinical outcome.

A randomized trial reported that high-amylose maize RS type 2 (HAM-RS2) reduced intrahepatic triglyceride content (IHTC) in adults enrolled under the former non-alcoholic fatty liver disease (NAFLD) definition [8]. Subsequent work connected variable response with baseline microbial features, competition among starch-utilizing organisms and experimental microbial rescue [9]. These findings bring a clinical question into focus: how can a defined RS exposure produce more consistent hepatic benefit, and can microbial information help select the most useful nutritional intervention for an individual?

This review connects the clinical evidence to three interacting determinants of response: substrate structure, microbial functional networks and host context. Earlier syntheses established RS structural diversity, variable microbial effects and the promise of personalized fiber interventions [6,10,11,12]. We build on that foundation by identifying the preparation and regimen with randomized hepatic-fat evidence, tracing the pathways that could connect its utilization to liver outcomes, and comparing findings with selected non-RS fibers. The evidence supports a specific HAM-RS2 research benchmark and an ecological explanation for response variation. The proposed path to precision nutrition tests whether measuring that ecology improves the choice between nutritional interventions.

## 2. Scope and Approach to the Evidence

This critical narrative review examines RS as a potential nutritional adjunct in adults with MASLD. Literature searches, updated through 11 October 2026, used PubMed-indexed records, Europe PMC and publisher websites with citation-led retrieval. Search terms covered resistant starch and high amylose in relation to MASLD, NAFLD, hepatic fat, microbiome, fermentation, response heterogeneity and precision nutrition. We identified human RS interventions with hepatic-fat measurements across countries and metabolic populations, together with mechanistic studies and selected non-RS fiber interventions.

Human RS interventions with hepatic outcomes anchored the clinical synthesis. Studies in other metabolic settings informed formulation specificity and transferability; culture, ex vivo and animal experiments informed mechanisms. Methodological publications guided interpretation of prediction and treatment–effect heterogeneity. Numerical findings were checked against the original publications and their accompanying supplementary materials, where available, with absolute percentage-point changes distinguished from relative changes. Shared participants were identified at the cohort level, and multicomponent diets were considered separately from RS supplementation. The review used targeted narrative selection, without duplicate screening, quantitative pooling or formal certainty grading.

## 3. What the Human Evidence Establishes

### 3.1. A Defined Nutritional Exposure Can Reduce Liver Fat

The strongest direct evidence is the four-month randomized, double-blind trial reported by Ni and colleagues [8]. Two hundred adults with NAFLD were assigned equally to HAM-RS2 or an energy-matched digestible-starch control; the reported primary analysis included 99 and 97 participants, respectively. The intervention supplied 40 g/day of RS within 91.2 g/day of preparation. The original key resources table identifies HI-MAIZE 260 (Ingredion Incorporated, Westchester, IL, USA; catalog 22000B00), consistent with the subsequent report [9]. Both groups received dietitian-led dietary and lifestyle counseling. In the original trial by Ni et al., IHTC was measured using proton magnetic resonance spectroscopy [8].The baseline-adjusted between-group difference in absolute IHTC change favored RS by 9.08 percentage points (95% confidence interval [CI], 6.26–11.91) [8].

Mean IHTC fell from 24.99% to 13.14% in the RS group and from 23.51% to 21.44% in the control group [8]. These correspond to absolute within-group decreases of 11.85 and 2.07 percentage points. Relative to each baseline group mean, the calculated reductions are 47.4% and 8.8%, respectively. These ratios of group means describe the magnitude of change; the trial’s 9.08-percentage-point estimate comes from its baseline-adjusted model. Thus, the absolute treatment contrast and the relative within-group reduction answer different questions.

Weight loss accompanied treatment. In a secondary analysis adjusting for weight change, the estimated difference remained 5.89 percentage points (95% CI, 2.91–8.87) [8]. The randomized comparison before adjustment for weight change remains the principal efficacy result. Because weight change occurs after randomization and may mediate benefit, the adjusted estimate is an exploratory indication of pathways beyond measured weight change; causal separation of those pathways requires additional assumptions and measurements.

The trial also connected hepatic improvement with microbial and serum metabolic changes, including lower branched-chain amino acids (BCAAs), and transfer experiments supported a microbial contribution [8]. Its clinical result applies to a defined HAM-RS2 exposure in adults enrolled with NAFLD and without diabetes, with liver fat as the measured endpoint. Replication in people with diabetes and trials incorporating fibrosis outcomes would extend this evidence to a broader MASLD population.

### 3.2. Response Variation Across Linked Cohorts

Long and colleagues revisited the RS-treated cohort and studied an additional multicenter, single-arm cohort [9]. Their high-response definition was at least a 30% relative reduction in IHTC. Trial 1 included 89 evaluable participants, of whom 67 were high responders and 22 low responders; Trial 2 included 55, of whom 35 were high responders and 20 low responders. The respective high-response proportions were 75.3% and 63.6%, calculated from the reported counts. Overall, 42 of 144 evaluable participants met the low-response definition. This descriptive proportion, 29.2%, applies to the selected treated cohorts.

The later report applied MASLD criteria to the parent cohort, excluding one participant who did not meet those criteria in addition to follow-up losses [9]. We therefore retain NAFLD for the parent trial’s enrollment and MASLD for the later reanalysis. Trial 2 added participants within the same research program and regional clinical setting, extending the assessment of response classification and prediction. Its single-arm design evaluates response under treatment; independent randomized replication addresses the treatment effect.

The linked cohorts also illustrate why the consumed exposure and measurement method deserve explicit reporting. Trial 2 used maize-derived HI-MAIZE 260 (Ingredion) at 78.4 g/day, supplying 40 g/day RS and a reported 153.7 kcal/day, with liver fat measured by magnetic resonance imaging-derived proton density fat fraction (MRI-PDFF) [9]. The parent trial used 91.2 g/day of powder, providing approximately 255 kcal/day, and proton magnetic resonance spectroscopy [8,9]. Reporting ingredient mass, analytically defined RS dose, energy and imaging method makes these cohort comparisons interpretable.

### 3.3. Interpreting Hepatic-Fat Response

Hepatic fat varies widely across MASLD populations. An MRS liver-fat level below 5% was used to define remission in a lifestyle trial [13], while imaging thresholds depend on the measurement method [2]. Against that reference, the Ni trial’s mean baseline of approximately 25% represents a substantial fat burden. A fall from 25% to 15% is a 10-percentage-point absolute decrease and a 40% relative decrease; a fall from 25% to 20% is a 5-percentage-point decrease and a 20% relative decrease. The observed fall to 13.14% therefore represents substantial improvement with residual steatosis. Histological inflammation, fibrosis and clinical events remain separate outcomes.

A meta-analysis of seven studies involving 346 adults with steatohepatitis associated a ≥30% relative reduction in MRI-PDFF with histological response, defined as a two-point improvement in NAFLD Activity Score, including improvement in inflammation or ballooning [14]. This supports the threshold as a candidate early-phase response measure. Baseline-adjusted continuous hepatic-fat outcomes preserve the magnitude of change, while a prespecified categorical measure complements clinical interpretation. Fibrosis improvement and longer-term liver outcomes require their own assessment.

The ≥30% relative-response threshold was already reached by 75.3% and 63.6% of evaluable RS recipients in the two Long cohorts [9]. These observations support a research aim of making substantial hepatic-fat responses more consistent. Independent trials can test whether matching the preparation to microbial function increases the proportion with sustained benefit. A reliable 60% relative reduction across recipients is a higher target requiring prospective evidence; it cannot be projected from the existing responder counts.

Observed response combines treatment effects with adherence, concomitant weight change, natural variation, regression to the mean and measurement error. These components help explain why a baseline feature associated with improvement among RS recipients may identify prognosis or realized exposure. Treatment selection asks a comparative question: does that feature identify greater benefit from RS than from an available alternative? Randomized comparisons and treatment-by-marker analyses connect the response phenotype to this decision [15].

Table 1 organizes hepatic outcomes by population, preparation and study independence. The Ni and Long reports describe a randomized NAFLD trial and linked response studies; British, American and Danish trials extend the hepatic evidence to other metabolic settings. Selected non-RS fiber trials place these findings within the wider context of fermentable substrates.

### 3.4. Hepatic and Metabolic Evidence Across Nutritional Settings

International RS trials have measured hepatic fat independently of the Ni–Long program. In Britain, Johnston et al. randomized 20 insulin-resistant adults with metabolic syndrome to HI-MAIZE 260 supplying 40 g/day RS or an available-carbohydrate-matched placebo for 12 weeks; 18 underwent MRI/MRS. Insulin sensitivity improved, while liver fat showed no significant treatment effect [16]. Bodinham et al. studied 17 adults with well-controlled type 2 diabetes in a 12-week crossover trial of HI-MAIZE 260 supplying 40 g/day RS. Among 14 participants with imaging, post-period liver fat was 9.7 ± 2.7% after RS and 10.0 ± 3.3% after control (mean ± SEM), with no significant difference [17]. The American STARCH trial randomized 68 adults with overweight or obesity and prediabetes to HAM-RS2, reported as 45 g/day, or isocaloric amylopectin for 12 weeks. Its overall analysis included 59 participants; the reported intrahepatic-lipid change contrast was −1.34 ± 3.96 on the authors’ IHL% scale (mean ± SD; *p* = 0.23) [18].

A Danish crossover trial included 19 completers with metabolic syndrome, with MRS measurements in 18. Four-week intervention diets combined cereal fiber, arabinoxylan and RS: the key foods supplied 21 g/day RS and 16 g/day arabinoxylan, versus 3 and 4 g/day in the refined-carbohydrate diet. Mean intrahepatic lipid was 17.0% to 16.9% with the fiber-rich diet and 16.9% to 16.9% with control; the between-diet change contrast was nonsignificant (*p* = 0.93) [19]. This multicomponent intervention informs dietary context, while RS-specific attribution requires an isolated contrast. The international findings establish a wider hepatic evidence base, with the positive randomized result in a NAFLD-enrolled population currently anchored by Ni et al. Differences in initial liver fat, metabolic disease, background intake and duration define candidate modifiers for independent MASLD trials.

Studies in other metabolic settings broaden the evidence on RS activity and formulation specificity. Wheat-derived native resistant granules (RS2) affected postprandial glycemia [25]; RS-containing potatoes and high-amylose wheat foods were tested within distinct food matrices [26,27,28]. Insulin secretion improved in a small trial [29]. In an obesity crossover trial, maize-derived HAM-RS2 (HI-MAIZE 260, Ingredion) was linked to weight loss and experimental evidence involving *Bifidobacterium adolescentis* [30]. Together, these studies show how the same broad substrate category can engage different metabolic outcomes across preparations and populations.

Other interventions produced smaller or null effects. RS and polydextrose had no overall effect on broad microbiota and metabolite profiles in a factorial study [31], and another RS crossover trial reported largely null cardiometabolic results [32]. A small metabolic-risk pilot found selected within-arm increases in adiposity or lipids, with corresponding between-group differences in change unestablished [33]. Interpreting this variation requires the preparation, comparator and endpoint to be considered together.

Variation across these studies directs attention to the sequence from substrate utilization to host response. Measuring both steps can identify whether an intervention achieves effective fermentation and whether that fermentation changes the selected clinical outcome.

### 3.5. Hepatic Outcomes with Non-RS Fermentable Fibers

Comparing RS with other fibers helps test how substrate properties shape hepatic benefit. Inulin-type fructans, arabinoxylans and other nondigestible carbohydrates differ in fermentability, viscosity, botanical matrix and dose [6,34]. These properties influence both the microbial process and the nutritional exposure.

Clinical findings show the importance of those distinctions. In a randomized pilot involving 14 adults with biopsy-confirmed NASH, Orafti P95 oligofructose (BENEO-Orafti S.A., Tienen, Belgium) improved histological steatosis relative to placebo after 36 weeks; total NAS declined significantly within the prebiotic arm, and fibrosis was unchanged [21]. In contrast, a 12-week inulin-type-fructan pilot in 19 adults found no significant liver-fat improvement despite increased *Bifidobacterium* abundance [22].

A Canadian 24-week trial randomized 43 adults with MASLD to oligofructose-enriched inulin (Synergy1, BENEO GmbH, Mannheim, Germany; 16 g/day) or isocaloric maltodextrin, with ten individualized dietitian sessions in both groups [23]. The reported intention-to-treat analysis included 42 participants and used last-value-carried-forward imputation. MRI liver-fat changes were −1.35 ± 3.51 versus −1.49 ± 3.57 percentage points (mean ± SD; *p* = 0.899); the per-protocol comparison was also nonsignificant (*p* = 0.866), as were FibroScan and FibroTest changes. Secondary analyses found a greater reduction in trunk-fat percentage and increased *Bifidobacterium* abundance with prebiotic supplementation. Thirty-two participants completed the trial, and weight reductions were modest. Attrition and imputation limit precision, while the hepatic results address the added value of this preparation alongside counseling.

In 18 adults with NAFLD, 42 days of inulin-propionate ester (IPE) versus inulin produced a nonsignificant between-group difference in liver-fat change (*p* = 0.082) [24]. Overall, these trials show mixed hepatic outcomes across substrates and settings. A direct comparison with HAM-RS2, using matched background care and the same hepatic endpoint, would test comparative benefit. The present cross-trial evidence supports that question without establishing equivalence or superiority.

### 3.6. Quantitative Comparison with Dietary and Lifestyle Treatment

Dietary and lifestyle treatment provides an effective background for evaluating an RS adjunct. The EASL–EASD–EASO guideline recommends sustained weight loss of at least 5% to reduce liver fat, 7–10% to improve liver inflammation and at least 10% to improve fibrosis in adults with overweight and MASLD [2]. These are body-weight targets. Dietary quality includes replacing saturated fats with unsaturated fats, limiting highly processed foods and sugary drinks, and adopting a Mediterranean-like pattern. Total fat reduction is one component of selected energy-restricted programs; fat quality and the overall dietary pattern also matter.

In Wong et al.’s randomized trial of 154 adults with NAFLD, a 12-month dietitian-led lifestyle program reduced MRS liver fat by 6.7 ± 6.1 percentage points, compared with 2.1 ± 6.4 percentage points under usual care (mean ± SD) [13]. Baseline mean liver fat was 12.3% and 12.2%, respectively. Remission, defined as liver fat below 5%, occurred in 64% versus 20% of participants. In the Look AHEAD ancillary study of 96 adults with type 2 diabetes, 12 months of intensive calorie restriction and physical activity produced a median relative hepatic-fat reduction of 50.8%, versus 22.8% with diabetes support and education [35]. The median absolute decreases were 2.3 and 1.1 percentage points, respectively. Baseline medians were 4.2% and 6.3%, lower than in the Ni trial, and 44% of this ancillary sample had NAFLD at enrollment.

Dietary composition can also affect hepatic fat. In a six-week randomized crossover study of 12 adults with biopsy-confirmed NAFLD, a Mediterranean diet rich in monounsaturated fats produced a mean relative MRS hepatic-fat reduction of 39%, compared with 7% on a low-fat, high-carbohydrate diet (*p* = 0.012), with no significant between-diet difference in weight loss [36]. At the end of the diet periods, fat supplied 44.3% of energy on the Mediterranean diet and 20.7% on the comparator; monounsaturated fats represented 51.0% and 39.0% of total fatty acids, respectively. The pattern change included food quality and fiber intake. This small trial illustrates why dietary composition belongs alongside energy balance when interpreting nutritional benefit.

The RS trial addresses added benefit within its own care setting: both groups received dietary and lifestyle counseling, and HAM-RS2 produced a 9.08-percentage-point greater liver-fat reduction than energy-matched digestible starch [8]. The calculated 47.4% reduction relative to the RS group’s baseline mean is within the range of substantial responses reported in lifestyle and dietary-composition studies. Cross-trial values provide clinical context; populations, initial fat burden, durations and summary statistics differ, so they cannot rank the interventions. The randomized RS contrast supports supplementation alongside the counseling used in that trial. Testing RS against a matched control within a sustained, optimized lifestyle program would establish its incremental benefit in that setting.

## 4. From Ingested Starch to a Microbial Nutritional Exposure

### 4.1. Structure Determines Substrate Accessibility

The five conventional RS classes describe mechanisms of resistance: physically inaccessible starch (RS1), native resistant granules (RS2), retrograded starch (RS3), chemically modified starch (RS4) and amylose–lipid complexes (RS5) [6]. RS1 occurs in intact or incompletely milled grains and pulses; RS2 includes high-amylose maize and native starch from sources such as potato or green banana; RS3 forms after gelatinized starch is cooled. RS4 and RS5 involve chemical modification and starch–lipid complex formation, respectively. Botanical source and processing further specify the exposure within each class. The hepatic trials examined maize-derived HAM-RS2, allowing their findings to be linked to a defined preparation.

Human experiments support this structural specificity. RS2 and RS4 produced different, reversible microbiota changes in a crossover study [37]. A later trial connected response variation to habitual fiber intake, baseline community features and treatment order [38], while shotgun metagenomics resolved species, strains and carbohydrate-utilization functions in samples from that same trial [39]. The two reports offer complementary views of one intervention population. Discrete RS4 structures also shifted fermentation toward different SCFAs in a human dose–response study [40], linking chemical structure to metabolic output.

Experimental systems make the exposure problem even clearer. Donor microbiomes fermented different starch sources with different butyrate yields [41]; resistant glucans with distinct linkages elicited different community and metabolite responses [42]. Preparation methods changed structure and fermentation characteristics in vitro [43], while crystallite stability influenced microbial interaction with RS5 [44]. Together, these studies show why preparation structure belongs in treatment-matching research. Equal nominal doses can provide different functional guilds with unequal amounts of accessible substrate.

### 4.2. Processing and Food Matrix Belong in the Intervention Definition

Isolated starch powders, whole grains, bread and cooked foods should not be treated as equivalent exposures. Their RS content coexists with different amounts of digestible carbohydrate, non-RS fiber, protein and other components. A high-amylose wheat study combined human and in vitro experiments [28]. Responses to the total fiber fraction differed from those to separated fractions. Most human secondary outcomes were unchanged, although *p*-cresol changed during the intervention. Attribution to RS alone is difficult when the food matrix changes several exposures simultaneously.

A reproducible intervention description should include the RS class and full name, botanical raw material, commercial product, manufacturer, batch specification and preparation as consumed. Production, storage, cooking or cooling, the RS assay, powder mass, available carbohydrate, energy and actual intake should also be reported. The linked MASLD cohorts illustrate why these details matter even when nominal RS doses are equal [8,9]. Structural and fermentation studies provide the rationale for characterizing the consumed preparation [6,40,43,44].

The comparator determines the nutritional question. An energy-matched digestible-starch control estimates the effect of replacing a specified exposure. Usual dietary advice provides a comparator for adding a program to care, while another fiber tests comparative substrate choice. In MASLD, the choice also depends on whether RS replaces other calories or supplements the habitual diet. Food displacement and adherence may contribute to the outcome. Dietary monitoring should capture those changes rather than assume that an assigned supplement dose describes the entire intervention.

### 4.3. Dose, Adaptation and Tolerance Can Modify the Realized Exposure

Higher dose does not necessarily imply a larger or more useful host effect. Dose–response work with defined RS4 preparations demonstrated structure-specific fermentation patterns [40], while a human study of other purified fibers found distinctive and individualized responses to different supplements [34]. In that study, the highest long-chain inulin dose increased inflammatory markers, and some participants showed alanine aminotransferase elevations [34]. These findings concern inulin rather than RS, but illustrate why dose-ranging studies should evaluate host outcomes and tolerability alongside fermentation. Adaptation, food displacement and gastrointestinal symptoms can influence the amount actually consumed and the duration over which it is sustained.

Tolerance helps determine the realized exposure. Long and colleagues reported no serious adverse events in Trial 2, alongside constipation, diarrhea, flatulence and increased intestinal gas passage [9]. Symptom trajectories, dose reductions, missed servings and discontinuation reasons can explain how laboratory fermentation potential translates into sustained intake. Longer follow-up would establish the acceptability of the large powder quantities used in these four-month trials.

## 5. Microbial Networks and Context-Dependent Utilization

### 5.1. Primary Degradation Is a Community Access Function

The ecological starting point is access to the starch particle. *Ruminococcus bromii* can initiate degradation of resistant substrates and release products accessible to organisms that cannot efficiently attack the same material alone [45]. Human dietary interventions associated *R. bromii*-related organisms with RS intake [46]. In a controlled feeding study of 14 overweight men, two participants had fecal recovery of more than 60% of ingested RS [47]. Recovery was below 4% in the others. The two participants with high recovery had low abundance of *R. bromii*-related organisms. The finding connects baseline community composition with substantial differences in substrate utilization.

Structural evidence explains why organism detection is only a partial functional measure. *R. bromii* organizes starch-binding and hydrolytic components in an extracellular amylosome, with spatial relationships supporting particle engagement and degradation [48]. Metagenomics identifies potential machinery; its activity also depends on expression, particle contact and intestinal conditions. Functional interpretation therefore requires the organism, substrate and surrounding community to be considered together.

Candidate degraders can be assessed through abundance, functional potential and activity relevant to the consumed preparation. Detection depends on sampling and assay sensitivity, while sufficient degradation depends on capacity at the assigned dose. Comparing these measurements with habitual diet and host phenotype can identify the most informative and practical test for treatment-matching research.

### 5.2. Cross-Feeding and Competition Shape the Output of Degradation

Primary degradation opens a resource to a network of consumers. Organisms may exchange oligosaccharides, organic acids and other fermentation products, while competitors alter access and growth. Metaproteomic work with individual microbiomes exposed to RS2, RS3 and RS4 found formulation-specific responses distributed across taxa and functions [49]. Ex vivo experiments with multiple RS preparations and microbiomes derived from inflammatory bowel disease similarly showed that donor identity could account for substantial response variation [50]. These experiments reveal community-dependent fermentation in inflammatory bowel disease, providing a mechanistic comparison for studies in other host settings.

Long and colleagues linked *Prevotella*-associated low response with inhibition of candidate RS degraders and identified *Bifidobacterium pseudocatenulatum* RRP01 as an experimental rescue organism [9]. Their human associations were complemented by co-culture, transfer and mouse interventions. This experimental perturbation supports a modifiable response network. Human translation now requires evaluation of RRP01 dose, persistence, safety and hepatic effects in the intended recipient population.

A parallel study in obesity connected RS response with *B. adolescentis* and experimental transfer and colonization evidence [30]. The studies implicate different species and strains within distinct communities and clinical outcomes. Rational microbial supplementation would need to demonstrate the function missing from the recipient ecosystem and show that the administered organism restores it under realistic dietary conditions. The ISAPP synbiotic framework defines combinations of live microorganisms and selectively utilized substrates [51]. Clinical evaluation must address each proposed combination and its intended benefit.

### 5.3. Prevotella Is a Test of Context, Resolution and Measurement

*Prevotella* illustrates why a universal “beneficial” or “unfavorable” label is unsuitable. Its association with lower HAM-RS2 response in the MASLD program contrasts with favorable glucose responses after barley-kernel bread and greater weight loss during whole-grain intake in other populations [9,52,53]. The studies differ in foods, phenotypes, outcomes and taxonomic resolution, providing several plausible sources of the divergent associations. Moreover, the older *P. copri* designation encompasses diversity now resolved within the *Segatella copri* complex, which has been extended to multiple species with distinct genomic features [54]. We retain the names used in the cited interventions to preserve traceability, while recognizing that genus-level markers combine biologically different organisms.

Absolute abundance and experimental interaction assays sharpen this ecological interpretation. Quantitative microbiome profiling showed that some taxonomic trade-offs in relative-abundance data reflect compositional effects [55]. Co-abundance networks identify candidate interactions, and competition assays test how those interactions affect resource use. The experimental components of the MASLD program connect these two levels; extending them to other *Prevotella* lineages and diets would test how widely the observed competition operates.

Broader human evidence reinforces the importance of baseline community and substrate context. Age and product context influenced microbial responses in older and middle-aged adults [56]; a small crossover study found different fiber-associated fermentation patterns in *Prevotella*- and *Bacteroides*-dominated microbiota [57]. Banana flour produced prominent microbial changes in a *Prevotella*-rich subgroup [58], and multi-cohort reanalysis found no single universally enriched beneficial species [59]. These findings support substrate-specific functional hypotheses for explaining response variation.

### 5.4. Host Physiology Changes the Ecological Setting

The host supplies more than a diagnostic label. Habitual fiber intake and intestinal physiology determine which resources reach the microbiota and how long they remain available. Stool consistency is associated with richness and community composition [60], while intensive human sampling linked gut physiological conditions, including transit and pH, with microbial and metabolic variation [61]. These observations support measuring intestinal context during biomarker development. Stool consistency offers a practical contextual measure, while direct transit assessment addresses a more specific physiological question.

Baseline taxonomy can also encode diet, medication use and metabolic phenotype. Small RS studies linked baseline microbiota to hormonal responses and internally predicted postprandial glycemia [62,63]. Evaluating such signals across diabetes status, fibrosis severity, concurrent metabolic treatment and dietary patterns would identify the settings in which they remain informative. For MASLD, combining these host measures with microbial function provides a way to distinguish biological dependencies from contextual predictors.

**Figure 1:**
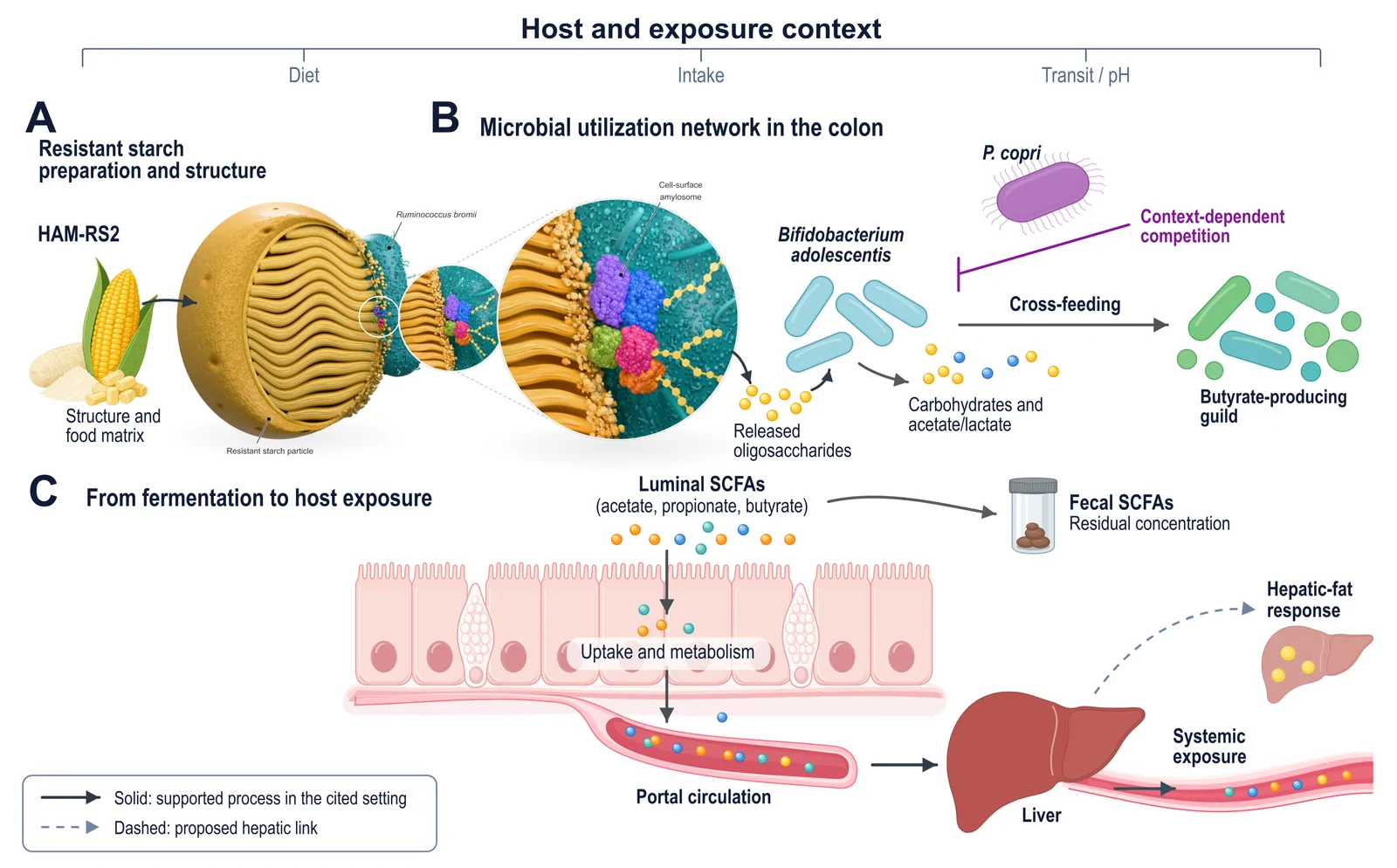
A measurement-aware framework connecting resistant-starch exposure to hepatic response. (**A**) The consumed HAM-RS2 preparation and its structure and food matrix define the nutritional exposure. The granule cutaway and molecular complex are schematic representations, not measured structures or microscopy. (**B**) *Ruminococcus bromii* and its cell-surface amylosome illustrate access to resistant-starch particles. Released carbohydrates support downstream utilization, including substrate-dependent activity of *Bifidobacterium adolescentis*, and cross-feeding involving carbohydrate intermediates, acetate and lactate. The butyrate-producing guild is a functional group rather than a single universal taxon. The *P. copri* competition symbol represents context-dependent interactions reported in the cited experimental systems; it does not assign a common inhibitory phenotype to the *Segatella copri* complex. (**C**) Fecal SCFAs represent residual luminal concentrations. Epithelial uptake and metabolism, portal delivery, hepatic handling and systemic exposure are distinct processes and compartments. Solid arrows depict supported processes in the cited settings; the dashed hepatic-response arrow denotes an indirect or incompletely established link to human hepatic response. Dotted magnification guides are graphical connectors, not biological interactions. Diet, actual intake, transit and pH condition these relationships. Colors are used schematically to distinguish the illustrated components rather than to indicate quantitative differences. In panel B, purple highlights P. copri and its context-dependent competition, light blue depicts B. adolescentis, and green/teal depicts the butyrate-producing guild. Molecules, organisms and lipid droplets are illustrative, with no quantitative scale [6,8,9,45,48,54,55,60,61,64]. HAM-RS2, high-amylose maize resistant starch type 2; SCFA, short-chain fatty acid.

Figure 1 brings these observations together as a sequence from the consumed preparation to microbial utilization and host exposure. Substrate, microbial network and host context jointly shape that sequence and its potential hepatic consequences.

## 6. Metabolic Readouts and the Evidence for Mediation

### 6.1. SCFA Absorption and Hepatic Metabolism

Colonic fermentation produces acetate, propionate and butyrate, whose absorbed fractions have different metabolic fates. Acetate can supply acetyl-CoA for oxidation or lipid synthesis; propionate can contribute carbon to hepatic gluconeogenesis; much of the butyrate is used before reaching peripheral blood. A stable-isotope study delivering labeled SCFAs to the colon of healthy adults estimated systemic availability at 36% for acetate, 9% for propionate and 2% for butyrate [64]. These peripheral estimates reflect intestinal and hepatic handling. Their differences show why interpreting RS fermentation requires attention to metabolite identity and metabolic fate.

SCFAs also engage intestinal endocrine pathways. In experimental systems, acetate and propionate stimulated glucagon-like peptide-1 release through free fatty acid receptor 2 [65]. This provides a route through which fermentation could alter appetite, insulin responses and substrate delivery to the liver, alongside direct hepatic carbon metabolism.

The direction of hepatic change depends on that metabolic balance. In the IPE-versus-inulin pilot, liver fat increased within the inulin arm, while the between-group comparison remained nonsignificant [24]. The authors proposed that propionate could counter an acetate-related lipogenic contribution. This hypothesis illustrates the need to follow individual SCFAs through host metabolism when testing an RS mechanism.

Fecal SCFAs measure the residual luminal pool after production, uptake and utilization. The randomized NAFLD trial measured ten fecal SCFAs and detected no differences after intervention despite the liver-fat benefit [8]. Human RS supplementation also produces variable fecal butyrate responses [66]. Higher concentrations can reflect increased production, reduced uptake or altered transit, whereas stable concentrations can accompany greater production and utilization. Stool water content and intestinal conditions contribute further variation [60,61]. Timed stool and circulating measurements, complemented by isotope tracing where feasible, would connect fermentation to absorbed host exposure.

The ecological framework therefore treats the SCFA profile, absorbed flux and host metabolism as successive links in the pathway to hepatic response. A mechanistic MASLD trial can test those links by combining a specified RS exposure with serial metabolite measurements and liver-fat assessment [8,9,64]. This design would identify which fermentation outputs are most useful for substrate selection.

### 6.2. BCAA Sources and Metabolic Context

Lower circulating BCAAs accompanied improvement in the HAM-RS2 trial, and later experiments linked impaired RS response to microbial and metabolic differences [8,9]. These findings support a candidate pathway connecting dietary substrate use with amino-acid metabolism. The contribution of this pathway is likely to depend on the preparation and host metabolic setting. Circulating concentration integrates dietary supply, microbial metabolism and host utilization, making it a biological state variable rather than a direct measure of bacterial production.

The distinction helps interpret apparently opposing animal findings. Oral BCAA supplementation improved steatosis in particular rodent settings, including experiments implicating microbiota-associated acetate production [67,68]. Supplemental exposure in a controlled model is not equivalent to an elevated endogenous concentration in human metabolic disease. Differences in dose, nutritional background, tissue use and microbial context can generate different outcomes. The productive next question is which part of BCAA metabolism changes with a defined RS preparation, and whether altering that part changes hepatic response.

### 6.3. Bile-Acid Handling and Lipid Absorption

Bile acids connect intestinal lipid digestion with enterohepatic recycling and metabolic signaling. Binding part of the luminal pool could increase fecal loss and reduce lipid or sterol absorption, while microbial transformation can change the signaling properties of the pool. These processes require distinct measurements. Most lipid absorption occurs in the small intestine, upstream of the principal site of RS fermentation, so a binding mechanism must be assessed for the consumed preparation in the relevant compartment. The randomized NAFLD trial detected modest changes in serum and fecal bile-acid profiles [8], motivating tests of bile-acid turnover alongside lipid absorption.

A static in vitro digestion study compared commercial Hylon VII (RS2), Novelose 330 (RS3) and Fibersym (RS4), alongside RS-containing foods [69]. The commercial preparations bound 6.3–10.0% of the amount bound by the cholestyramine positive control under the same assay conditions. Binding did not differ significantly among the tested food and RS samples, and neither RS content nor class predicted binding. This modest assay-specific capacity motivates product-specific testing. It is a ratio to the assay control, rather than an estimate of bile acids removed in vivo; HI-MAIZE 260 was not tested.

The obesity crossover trial provides a separate line of evidence on lipid handling [30]. In 17 participants with fecal lipid measurements, daily non-esterified fatty-acid, triglyceride and cholesterol excretion was higher after HAM-RS2 than after control starch despite similar dietary fat intake. The accompanying mouse studies implicated microbiota-dependent intestinal angiopoietin-like 4 (ANGPTL4), with increased fecal triglyceride and reduced intestinal lipase activity. These observations support an absorption-related pathway in that setting. Testing its contribution to MASLD would pair hepatic outcomes with fecal lipid measurements and bile-acid turnover; direct binding and microbiota-dependent enzyme regulation are separate candidate mechanisms.

The DISC trial provides further evidence for changes in bile-acid profiles. Participants consumed 23 g/day of maize-derived HI-MAIZE 260 (Ingredion), containing approximately 12 g/day of native resistant granules (RS2), with or without polydextrose [70]. Broad microbiota and metabolite analyses were largely null [31], while targeted analysis found increases in selected circulating conjugated bile acids and changes in fecal bile-acid proportions [70]. These profile measurements address composition and concentration. Total excretion, pool turnover and fecal lipids would address sequestration and absorption, while receptor-related measurements would clarify signaling.

### 6.4. Integrating the Candidate Pathways

Within the hepatic studies, microbiota-dependent amino-acid and inflammatory pathways have the strongest experimental support [8,9]. Reduced *Bacteroides stercoris* abundance and circulating BCAAs accompanied improvement in the randomized trial, and donor-microbiota transfer reproduced aspects of the phenotype in mice. Live and heat-killed *B. stercoris* experiments supported inflammatory and BCAA-related routes, while valine promoted lipid accumulation in HepG2 cells [8]. The later study added microbial competition and rescue experiments linking substrate utilization to response [9]. RS4 and combined arabinoxylan–RS studies broaden the immunometabolic context in other populations [20,71]. The relative contribution of these pathways to human hepatic benefit remains to be quantified. Serial measurements and comparative perturbations can distinguish SCFA flux, bile-acid recycling, lipid absorption, BCAAs and weight-related contributions. Table 2 aligns each measurement with the next mechanistic question.

## 7. What Recent Precision-Nutrition Studies Add

Recent precision-nutrition studies provide methods for connecting individual biology to dietary response. Postprandial glucose models combined clinical, dietary and microbial information [73], and evaluation in a US cohort tested transport beyond the development setting [74]. For MASLD, the same progression can link a clearly specified hepatic outcome to external evaluation and a nutritional decision.

Clinical comparisons show why the selected endpoint and default care pathway matter. A multicomponent personalized program improved triglycerides, with no significant effect on low-density lipoprotein cholesterol, its other primary endpoint [75]. Another randomized trial found similar weight loss with an algorithm-guided diet and a low-fat diet [76]. These results make a practical comparison central to precision nutrition: how much benefit does microbial information add to accessible dietary and clinical assessment?

A trial of 802 adults with prediabetes illustrates the opportunity and the need for prospective evaluation [77]. Mixed fiber and usual care produced no significant overall difference in the primary glycated-hemoglobin outcome. Post hoc metabolic clusters and geographically concentrated microbial modeling suggested treatment-matching hypotheses. The primary endpoint had replaced a planned longer-term diabetes-progression outcome after pandemic-related disruption. Those exploratory signals provide candidates for a prospective comparison using a specified substrate and hepatic endpoint.

Functional screening offers a complementary approach. An ex vivo screen prospectively ranked fibers for eight healthy Japanese adults [72]. In a nonrandomized crossover comparison, the highest-ranked option favored fecal volatile fatty acids; significant participant-level increases versus baseline occurred in two of eight participants, and the assay differential did not predict response magnitude. This small study establishes a practical workflow from community testing to substrate allocation. Its extension to MASLD would link assay repeatability and substrate ranking to a hepatic endpoint and an implementable default.

Diet-wide fiber and fermented-food studies also produced different microbial and immune responses [78], consistent with structure-specific fiber effects [34]. Their practical contribution is a testable design principle: compare functional assays, taxonomy and simple clinical predictors using the same defined intervention and the same hepatic outcome.

## 8. From Response Prediction to a Treatment Decision

### 8.1. What the Existing Classifier Predicts

In the linked MASLD study, a model combining microbial and clinical features distinguished high from low observed hepatic-fat response [9]. Its area under the receiver operating characteristic curve (AUC) was 0.87 (95% CI, 0.83–0.92) in Trial 1 and 0.74 (95% CI, 0.66–0.81) in Trial 2. These estimates describe response ranking among treated participants and provide a starting point for external evaluation. Comparative intervention data would connect the prediction to a choice between RS and another nutritional option.

The development analysis included 89 participants and used outcome-informed feature selection, model tuning and class balancing [9]. Reproduction should document how these steps were confined to development data and nested during resampling. Evaluation in larger independent cohorts would then estimate performance across dietary and clinical settings.

TRIPOD+AI and PROBAST+AI provide reporting and appraisal frameworks [79,80]. A reproducible test requires fixed specimen handling, feature definitions, preprocessing, model parameters and rules for thresholds or missing data.

### 8.2. Calibration and Practical Value

Calibration complements ranking by testing whether predicted probabilities agree with observed outcomes [81]. The inspected MASLD report presented discrimination estimates, with calibration, prediction error and decision benefit left for further evaluation [9]. Independent testing can assess these properties and establish whether recalibration is needed for the intended population.

Clinical-only, diet-and-clinical and microbiome-augmented models should be compared on the same participants and endpoint. Accuracy, assay failure, cost and turnaround time matter because a test is useful only if its information changes a feasible decision.

Decision-curve analysis can summarize the consequences of thresholds when the action, comparator and outcome costs are specified [82]. For a decision to switch from RS to another substrate, comparative intervention data supply the expected outcomes under each option. Combining those data with decision analysis connects prediction accuracy to a feasible clinical choice.

### 8.3. Identifying Differential Treatment Benefit

A prognostic marker identifies people likely to improve or remain at risk under a defined care pathway. A treatment-selection marker identifies variation in the comparative effect of available options. Estimating the latter requires treatment assignment and appropriate interactions, as emphasized by the PATH framework [15]. For RS, the comparison could be HAM-RS2 versus a characterized alternative substrate, or a matched microbial adjunct versus placebo with the same RS exposure.

A marker-stratified trial would randomize participants to competing interventions within prespecified marker strata and directly test the marker-by-treatment interaction. Keeping the marker continuous where feasible preserves information; prespecified categories can support operational decisions. Power should address the interaction and its uncertainty, allowing the trial to determine whether the marker distinguishes comparative benefit.

### 8.4. Defining Response for Clinical Use

A clinically useful response definition specifies intervention, outcome, time and measurement. Low hepatic response to one HAM-RS2 regimen can coexist with fermentation responses to another substrate or benefits in other endpoints. Actual intake and concomitant weight change help interpret that phenotype. Reporting “low hepatic-fat response to this HAM-RS2 regimen” preserves the context needed for a subsequent nutritional choice.

This also affects implementation. If the assay fails or the prediction is outside the population represented in development, an accessible default care pathway is needed. The default pathway should preserve access to nutritional counseling and metabolic care. Evaluation can then establish whether microbial testing provides sufficient additional benefit to justify its use.

## 9. A Research Agenda with Explicit Decision Points

### 9.1. Establish Reproducibility at the Exposure and Assay Levels

The tested HAM-RS2 regimen provides a concrete benchmark for independent replication. In the parent trial, HI-MAIZE 260 (Ingredion; catalog 22000B00) supplied 40 g/day RS in 91.2 g/day powder for 120 days, with reported energy of 255.4 kcal/day; the control supplied 72 g/day digestible starch and 255.6 kcal/day [8]. Both groups received dietitian-led dietary and lifestyle counseling. Three-day dietary records and returned empty packets assessed adherence, and radiologists measuring hepatic triglyceride by proton magnetic resonance spectroscopy were blinded to clinical information. No serious adverse events were reported; increased intestinal gas passage occurred in 35 RS recipients and eight control recipients. Replication across geography, background diet and clinical phenotype, followed by dose-ranging and preparation comparisons, would identify which exposure produces the most consistent benefit.

To establish added benefit under contemporary nutritional care, an independent trial could provide the same structured dietary-quality, energy-management and physical-activity program in both arms, then randomize HAM-RS2 versus a matched control. Prespecified baseline hepatic-fat and diabetes strata would test transferability across the contrasting metabolic populations in Table 1. This design connects efficacy replication to the practical question of what RS adds to an effective care pathway.

In parallel, repeated baseline testing can establish the reproducibility of candidate microbial and fermentation assays. Specimen handling, storage, assay batches and the consumed substrate should be specified. Comparing each assay with dietary and clinical measures would identify its added information and guide refinement before a treatment-matching trial.

### 9.2. Compare Defined Treatment-Matching Options

A first comparative trial should answer one matching question cleanly. For substrate matching, participants could be randomized within marker strata to HAM-RS2 or a structurally characterized alternative, with both groups receiving equivalent background counseling. For microbial rescue, a factorial design could compare RS and control substrate with and without a defined candidate organism, if prior human safety and manufacturing requirements are satisfied. The latter design can distinguish effects of the organism alone from effects of its combination with RS. RRP01 provides a mechanistic candidate for this staged evaluation [9,51].

The primary estimand should be prespecified. An intention-to-treat comparison captures assignment under realistic adherence, while a separately planned adherence-sensitive analysis may explore realized exposure. Baseline-adjusted continuous liver-fat change, measured by a harmonized blinded protocol, is a defensible early efficacy endpoint. The 30% relative-response threshold can be secondary. Fibrosis-related measures, metabolic outcomes, gastrointestinal symptoms and sustained intake should be reported as distinct outcomes. Longer follow-up and fibrosis-related endpoints would clarify whether early hepatic-fat changes translate into sustained disease benefit.

### 9.3. Locate the Mechanisms That Shape Response

The three-determinant framework should generate competing predictions. If inadequate degradation is the limiting step, a matched preparation or degrader intervention should improve substrate utilization before a host benefit emerges. If utilization improves without hepatic change, the limiting step may lie downstream in host exposure or hepatic responsiveness. If a taxonomic marker loses predictive value after careful dietary or transit assessment, it may be a proxy rather than an indispensable biological selector. If clinical variables alone match the combined model, sequencing may be unnecessary even when microbiota participate mechanistically.

Serial sampling is essential to distinguish these possibilities. Baseline measurements characterize the recipient state; early measurements assess exposure and fermentation; later measurements assess systemic and hepatic effects. Mechanistic endpoints should be chosen in advance, with compartment and timing matched to the proposed link. Formal mediation analysis requires its own assumptions, and animal transfer can support biological plausibility without estimating the mediated fraction in humans. These observations would locate the step at which treatment matching improves, or fails to improve, hepatic response.

### 9.4. Evaluate the Strategy Against an Implementable Default

After a treatment-selection rule has been fixed, a strategy trial should compare marker-guided allocation with the best feasible unstratified dietary approach. This is distinct from the marker-stratified trial: it asks whether using the information improves outcomes at the service level. The comparator should receive equivalent counseling, contact and follow-up so that any advantage cannot be attributed simply to greater attention. The analysis should include assay cost, time to treatment, adherence, symptoms, patient-reported acceptability and the consequences of unavailable or indeterminate results.

**Figure 2:**
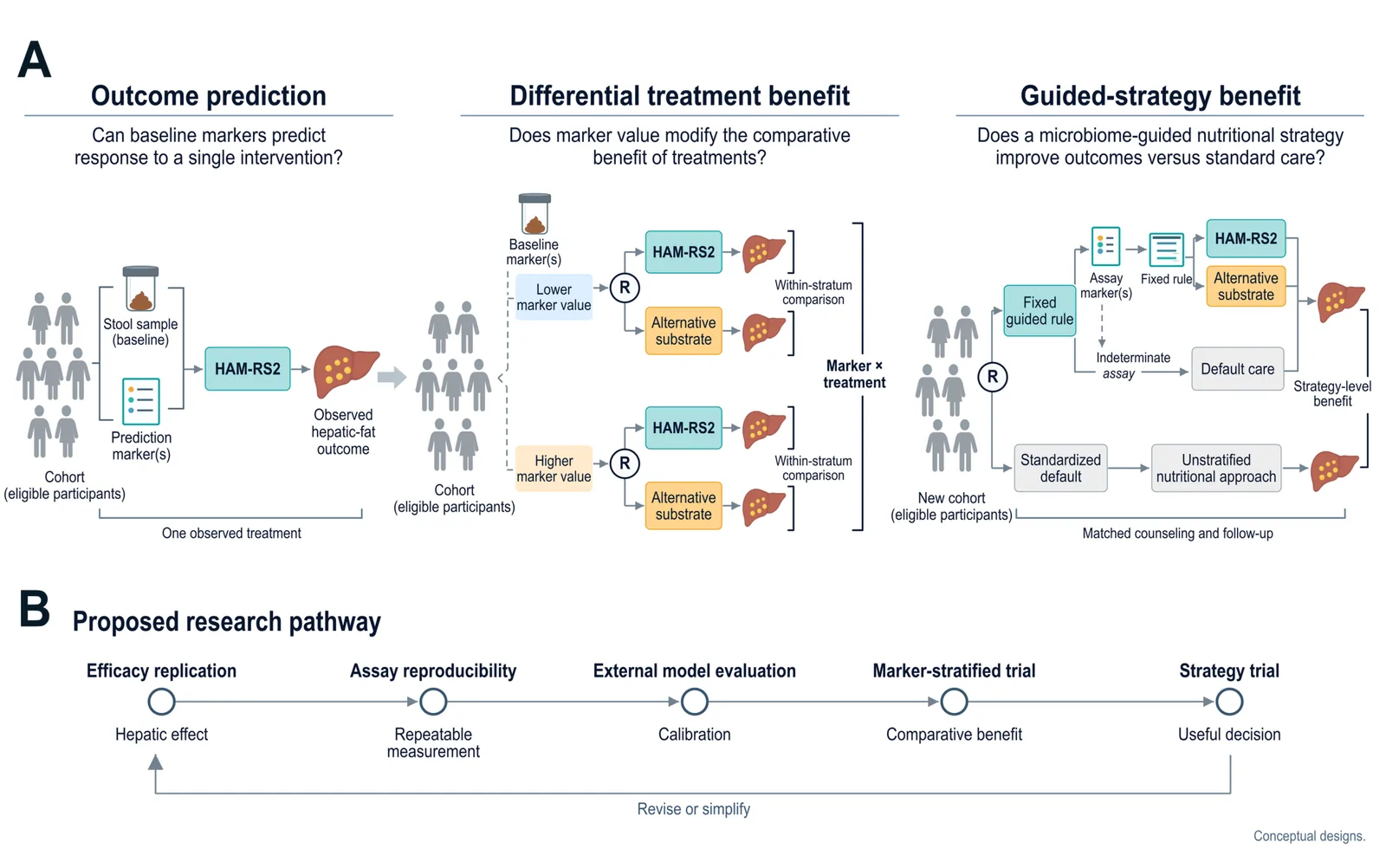
From response prediction to evidence that microbiome-guided nutrition improves outcomes. (**A**) Outcome prediction concerns the observed hepatic-fat outcome during one specified intervention. A marker-stratified trial instead randomizes participants to both nutritional alternatives within each marker stratum and compares the treatment contrasts across strata. The two strata are illustrative; continuous-marker analyses can also be used. A separate strategy trial randomizes a new cohort to a fixed guided rule or a standardized default, with matched counseling and follow-up. Participants with indeterminate assays follow a fallback pathway within the guided strategy and remain part of its evaluation. R denotes randomization. The transition between motifs represents a change in research question, not transfer of participants between completed trials. Liver symbols and participant counts are schematic and encode no outcome magnitude or sample size. (**B**) The proposed pathway links independent efficacy replication, assay reproducibility, external model evaluation, a marker-stratified trial and a strategy trial. Progression depends on reproducibility, calibration, comparative benefit and useful decisions; findings that do not support progression prompt revision or simplification. The panels summarize proposed study designs [15,79,80,81,82]. HAM-RS2, high-amylose maize resistant starch type 2.

Longer follow-up should test whether benefit persists, whether a baseline assay remains informative after sustained dietary change, and whether repeated testing improves decisions enough to justify its burden. Equity is part of effectiveness: an algorithm trained in a narrow dietary and geographic setting can deliver unequal performance when transferred elsewhere. Evaluation across relevant subgroups should be planned, with uncertainty reported rather than inferred from underpowered subgroup comparisons. Table 3 and Figure 2 translate these priorities into a staged pathway with explicit evidence requirements.

## 10. Discussion

RS offers a tractable test of precision nutrition because the exposure can be characterized, microbial degradation can be probed and hepatic fat can be measured repeatedly. A defined HAM-RS2 regimen has randomized hepatic-fat evidence, while linked ecological studies identify processes that could explain variation in response. Together, these findings connect a modifiable dietary substrate to microbial resource use and host metabolic exposure. Its potential role in MASLD is as an adjunct to dietary quality, weight management and risk-appropriate metabolic care.

The three-determinant framework brings divergent findings into a coherent biological sequence. Substrate structure sets accessibility, community interactions shape utilization and host context determines metabolic consequences. *Prevotella* associations vary across foods and phenotypes; BCAA findings depend on the source and setting of exposure; fecal SCFAs capture the luminal residue of a dynamic metabolic process. Bile-acid and lipid-absorption evidence broadens the downstream pathways to test. Null hepatic findings in international RS trials and mixed results with other fibers emphasize that fermentation needs to be connected to a specified clinical outcome and recipient population.

Independent randomized replication in a MASLD-enrolled population remains the main clinical evidence gap. The international hepatic-fat studies identified here enrolled people with metabolic syndrome, diabetes or prediabetes, whereas the positive Ni trial enrolled adults with NAFLD without diabetes. This contrast gives clinical purpose to studying background diet, baseline hepatic fat, metabolic phenotype and effective substrate utilization. Short interventions, small samples and shared cohorts affect precision and transferability; the targeted narrative approach also limits completeness and formal certainty assessment. Wider geographic and clinical sampling, consistent exposure characterization and harmonized hepatic outcomes would establish where the observed benefit is reproducible.

Dietary control can strengthen that next step. Controlled feeding in residential settings, including care homes, military facilities or other institutions with standardized meals, could reduce variation in background intake when voluntary informed consent and representative recruitment are ensured. Complementary community trials would assess adherence and transfer under everyday conditions. Within these settings, the most useful predictor may be a focused functional panel, an ex vivo screen or routine clinical information combined with dietary assessment. Comparing these approaches against the same nutritional choice would establish which information improves outcomes at an acceptable burden.

## 11. Conclusions

A defined HAM-RS2 intervention has reduced liver fat in a randomized NAFLD trial alongside dietary counseling. International trials in other metabolic settings help define the context in which that finding should be replicated. Ecological evidence connects variation in response with substrate accessibility, microbial interactions and host context. This framework guides research on how a consumed preparation becomes a hepatic metabolic exposure and how that process can be modified. Independent replication and marker-stratified trials can establish whether measuring these features improves the choice between nutritional interventions. Evaluation of a fixed guided strategy would then connect the mechanistic promise of RS to clinical decisions alongside standard MASLD care.

## Figures and Tables

**Table 1:** Human hepatic outcomes with resistant starch and selected non-RS fermentable fibers.

Study and Population	Preparation and Design	Hepatic Information	Evidence Context
Ni et al. [8]; Chinese adults with NAFLD; diabetes excluded	Maize-derived native resistant granules (HAM-RS2); HI-MAIZE 260, Ingredion; catalog 22000B00; 91.2 g powder/day supplying 40 g RS; four months; 200 randomized; 196 in primary analysis	Mean IHTC 24.99% to 13.14% (RS) versus 23.51% to 21.44% (control); baseline-adjusted change contrast −9.08 percentage points (95% CI −11.91 to −6.26); MRS	Randomized added benefit alongside counseling; 47.4% relative decrease calculated from RS group means
Long et al., Trial 1 [9]; MASLD reanalysis	89 evaluable RS recipients from Ni et al.; same maize-derived HI-MAIZE 260 exposure; 67 high and 22 low responders	≥30% relative IHTC reduction in 67/89 (75.3%); microbial and experimental analyses of variation	Linked treated-cohort analysis of response heterogeneity
Long et al., Trial 2 [9]; Chinese adults with MASLD	HI-MAIZE 260, Ingredion; 78.4 g powder/day supplying 40 g RS; four months; single arm; 60 enrolled, 55 evaluable	≥30% relative hepatic-fat reduction in 35/55 (63.6%); measured by MRI-PDFF	Single-arm assessment of response in additional participants; powder mass, energy and imaging differ from the parent trial
Johnston et al. [16]; Britain; 20 adults with insulin resistance and metabolic syndrome; imaging *n* = 18	HI-MAIZE 260; 40 g RS plus 27 g rapidly digestible starch/day; Amioca placebo with 27 g digestible starch/day; single-blind randomized parallel trial; 12 weeks	MRS-measured hepatic fat: no significant treatment effect; insulin sensitivity improved	Independent of Ni–Long; a British metabolic-syndrome cohort with a hepatic endpoint
Bodinham et al. [17]; Britain; 17 adults with well-controlled type 2 diabetes; imaging *n* = 14	HI-MAIZE 260, Ingredion; 67 g powder/day supplying 40 g RS; available-carbohydrate-matched Amioca control; randomized crossover; 12-week periods	Post-period hepatic fat: RS 9.7 ± 2.7% versus control 10.0 ± 3.3% (mean ± SEM); nonsignificant	Related to the Johnston research program; separate cohort; liver fat reported after each period, rather than as an RS change score
Peterson et al. [18]; USA; 68 adults randomized with overweight or obesity and prediabetes; overall analysis *n* = 59	HAM-RS2, HI-MAIZE 260, reported as 45 g/day; isocaloric Amioca amylopectin control; double-blind randomized parallel trial; 12 weeks	MRS IHL change contrast −1.34 ± 3.96 on the reported IHL% scale (mean ± SD; *p* = 0.23); nonsignificant	Independent American STARCH trial; oil-normalized IHL scale; extreme IHL outliers excluded in the reported analysis
Schioldan et al. [19]; Denmark; 19 adults with metabolic syndrome completed; MRS *n* = 18	Key foods: 21 g RS and 16 g arabinoxylan/day versus 3 g RS and 4 g arabinoxylan/day; cereal-fiber-rich versus refined-carbohydrate diet; randomized crossover; four-week periods	MRS IHLC: 17.0 ± 10.1% to 16.9 ± 9.1% versus 16.9 ± 10.1% to 16.9 ± 10.1% (mean ± SD); change contrast *p* = 0.93	Independent Danish cohort; multicomponent diet; same intervention as Hald et al. [20]
Bomhof et al. [21]; 14 adults with biopsy-confirmed NASH	Non-RS oligofructose, Orafti P95 (BENEO-Orafti S.A., Tienen, Belgium); 8 g/day for 12 weeks then 16 g/day for 24 weeks, versus isocaloric maltodextrin	Between-group histological steatosis improvement; total NAS improved within the prebiotic arm; no fibrosis improvement	Pilot histological evidence for a different fiber, dose and duration
Reshef et al. [22]; 19 adults with NAFLD and metabolic syndrome	Non-RS inulin-type fructans, 16 g/day, versus maltodextrin for 12 weeks; weight-maintenance setting	No significant hepatic-fat improvement despite increased *Bifidobacterium* abundance	Pilot examining microbial and hepatic outcomes during weight maintenance
Mayengbam et al. [23]; Canadian adults with MASLD; 43 randomized, 42 in reported ITT analysis, 32 completed	Non-RS oligofructose-enriched inulin, Synergy1 (BENEO GmbH, Mannheim, Germany), 16 g/day, versus isocaloric maltodextrin, AGENAMALT 20.222 (AGRANA Stärke GmbH, Vienna, Austria); 24 weeks; ten dietitian sessions in both groups	MRI liver-fat change (mean ± SD): −1.35 ± 3.51 versus −1.49 ± 3.57 percentage points; ITT *p* = 0.899; PP *p* = 0.866; no between-group FibroScan or FibroTest improvement	Added-value comparison alongside counseling; attrition and last-value-carried-forward imputation affect precision
Chambers et al. [24]; 18 adults with NAFLD	IPE versus inulin, 20 g/day for 42 days; randomized parallel design	Between-group liver-fat-change comparison: *p* = 0.082; within-arm increase with inulin	Exploratory comparison of SCFA delivery with two non-RS preparations

NAFLD (non-alcoholic fatty liver disease) and NASH (non-alcoholic steatohepatitis)denote historical enrollment definitions. Countries refer to clinical study settings. Johnston and Bodinham represent separate cohorts within a related British research program; both are independent of the Ni–Long program. The Long Trial 1 cohort overlaps the Ni trial; Trial 2 enrolled additional participants. The Danish intervention changed RS and other dietary components together. Peterson’s IHL values retain the authors’ external-oil-normalized MRS scale. Imaging sample sizes are distinguished from total study samples. Non-RS trials are selected contextual comparisons. IHTC, intrahepatic triglyceride content; HAM-RS2, high-amylose maize resistant starch type 2; MRS, magnetic resonance spectroscopy; MRI-PDFF, magnetic resonance imaging proton density fat fraction; NAS, NAFLD Activity Score; IPE, inulin-propionate ester; MASLD, metabolic dysfunction-associated steatotic liver disease; ITT, intention-to-treat; PP, per protocol; SD, standard deviation; SEM, standard error of the mean; RS, resistant starch; CI, confidence interval; IHL, intrahepatic lipid; IHLC, intrahepatic lipid content; MRI, magnetic resonance imaging; SCFA, short-chain fatty acid.

**Table 2:** Measurements and mechanistic questions along the resistant-starch–microbiome–liver pathway.

Measurement Layer	Supported Interpretation	Question for Further Testing	Useful Next Measurement
RS content and preparation [6,40,42]	Characterizes the administered exposure and its processing history	How much of the consumed preparation becomes accessible to the colonic community?	Analyze RS in the consumed preparation; record matrix, processing, storage and actual intake
Taxon abundance and starch-degradation machinery [45,48,55]	Identifies candidate organisms, functional capacity and community context	How does detected capacity translate into activity and substrate utilization?	Absolute abundance, functional expression and directly measured substrate disappearance
Ex vivo fermentation [50,72]	Measures community behavior under a defined assay condition	How well does assay ranking predict in vivo hepatic response?	Repeatability, matched consumed substrates and prospective linkage to human outcomes
Fecal SCFAs [60,61,64,66]	Describes residual luminal concentrations at sampling	How do production, absorption and host use contribute to hepatic exposure?	Timed stool and blood sampling; isotope tracing to distinguish production, absorption and host use
Circulating BCAAs and compartment-specific bile acids [8,31,67,68,70]	Captures metabolic associations in the sampled blood or fecal compartment	Which metabolic changes mediate benefit, and what is their relative contribution?	Serial metabolic sampling; bile-acid pool or total excretion assessment; fecal fat or sterol measurements; causal tests
MRI-based liver fat and response categories [8,9,14]	Measures a hepatic-fat endpoint and an explicitly defined change	Does the hepatic-fat change accompany fibrosis improvement or identify comparative treatment benefit?	Independent randomized replication; appropriate fibrosis outcomes; prespecified marker-by-treatment interaction

The final column identifies measurements for mechanistic and treatment-selection research. BCAA, branched-chain amino acid; SCFA, short-chain fatty acid; RS, resistant starch; MRI, magnetic resonance imaging.

**Table 3:** Proposed stages and decision criteria for translating microbiome information into resistant-starch treatment selection.

Stage and Question	Essential Design	Evidence Permitting Progression	Action Following a Negative Result
1. Is the nutritional effect reproducible?	Independent randomized trial with a specified preparation, credible comparator, adherence and tolerability assessment	Clinically relevant hepatic-fat benefit with uncertainty, in a clearly defined population	Reassess the product, dose and population before developing a biomarker-based strategy
2. Is the candidate measurement dependable?	Repeated baseline samples; prespecified handling and assay procedures; functional testing where justified	Reproducibility and added information beyond accessible clinical measurements	Improve or simplify the assay before deploying a treatment-selection rule
3. Does prediction transport?	Freeze the complete model and threshold; evaluate in independent participants without retuning on their outcomes	Discrimination, calibration and error patterns adequate for the intended setting	Recalibrate or revise using separate data, then evaluate the updated model anew
4. Does the marker distinguish comparative benefit?	Randomize within marker strata; prespecify and power a marker-by-treatment interaction	Evidence that the marker identifies differential benefit relevant to a feasible nutritional choice	Use the marker for prognosis or mechanistic research; pursue other predictors of comparative benefit
5. Does using the rule improve care?	Randomize a locked guided strategy against an implementable default; include assay failures and adherence in strategy-level analysis	Better outcomes or a justified benefit–burden trade-off including cost, access and tolerability	Prefer the simpler strategy or redesign the decision pathway

This proposed pathway draws on prediction and treatment–effect heterogeneity methodology [15,79,80,81,82]. Progression thresholds should be prespecified for the population, endpoint and practical decision being evaluated.

## Data Availability

No new data were created or analyzed in this study. Data sharing is not applicable to this article.

## References

[1] Rinella M.E., Lazarus J.V., Ratziu V., Francque S.M., Sanyal A.J., Kanwal F., Romero D., Abdelmalek M.F., Anstee Q.M., Arab J.P. (2023). A multisociety Delphi consensus statement on new fatty liver disease nomenclature. Hepatology.

[2] European Association for the Study of the Liver (EASL), European Association for the Study of Diabetes (EASD), European Association for the Study of Obesity (EASO) (2024). EASL-EASD-EASO Clinical Practice Guidelines on the Management of Metabolic Dysfunction-Associated Steatotic Liver Disease (MASLD). Obes. Facts.

[3] Harrison S.A., Bedossa P., Guy C.D., Schattenberg J.M., Loomba R., Taub R., Labriola D., Moussa S.E., Neff G.W., Rinella M.E. (2024). A Phase 3, Randomized, Controlled Trial of Resmetirom in NASH with Liver Fibrosis. N. Engl. J. Med..

[4] Sanyal A.J., Newsome P.N., Kliers I., Østergaard L.H., Long M.T., Kjær M.S., Cali A.M., Bugianesi E., Rinella M.E., Roden M. (2025). Phase 3 Trial of Semaglutide in Metabolic Dysfunction–Associated Steatohepatitis. N. Engl. J. Med..

[5] Lau H.C.-H., Zhang X., Yu J. (2025). Gut microbiome in metabolic dysfunction-associated steatotic liver disease and associated hepatocellular carcinoma. Nat. Rev. Gastroenterol. Hepatol..

[6] Dobranowski P.A., Stintzi A. (2021). Resistant starch, microbiome, and precision modulation. Gut Microbes.

[7] Gibson G.R., Hutkins R., Sanders M.E., Prescott S.L., Reimer R.A., Salminen S.J., Scott K., Stanton C., Swanson K.S., Cani P.D. (2017). Expert consensus document: The International Scientific Association for Probiotics and Prebiotics (ISAPP) consensus statement on the definition and scope of prebiotics. Nat. Rev. Gastroenterol. Hepatol..

[8] Ni Y., Qian L., Siliceo S.L., Long X., Nychas E., Liu Y., Ismaiah M.J., Leung H., Zhang L., Gao Q. (2023). Resistant starch decreases intrahepatic triglycerides in patients with NAFLD via gut microbiome alterations. Cell Metab..

[9] Long X., Wang H., Lu Y., Gao X., Xiao Y., Zhang M., Guo J., Yang J., Zhang R., Li Q. (2025). Interindividual variability in gut microbiome mediates the efficacy of resistant starch on MASLD. Cell Metab..

[10] Chen R., Zhang C., Xu F., Yu L., Tian F., Chen W., Zhai Q. (2023). Meta-analysis reveals gut microbiome and functional pathway alterations in response to resistant starch. Food Funct..

[11] Bendiks Z.A., Knudsen K.E.B., Keenan M.J., Marco M.L. (2020). Conserved and variable responses of the gut microbiome to resistant starch type 2. Nutr. Res..

[12] Kok C.R., Rose D.J., Hutkins R. (2023). Predicting Personalized Responses to Dietary Fiber Interventions: Opportunities for Modulation of the Gut Microbiome to Improve Health. Annu. Rev. Food Sci. Technol..

[13] Wong V.W.S., Chan R.S.M., Wong G.L.-H., Cheung B.H.K., Chu W.C.W., Yeung D.K.W., Chim A.M.L., Lai J.W.Y., Li L.S., Sea M.M.M. (2013). Community-based lifestyle modification programme for non-alcoholic fatty liver disease: A randomized controlled trial. J. Hepatol..

[14] Stine J.G., Munaganuru N., Barnard A., Wang J.L., Kaulback K., Argo C.K., Singh S., Fowler K.J., Sirlin C.B., Loomba R. (2021). Change in MRI-PDFF and Histologic Response in Patients With Nonalcoholic Steatohepatitis: A Systematic Review and Meta-Analysis. Clin. Gastroenterol. Hepatol..

[15] Kent D.M., Paulus J.K., van Klaveren D., D’AGostino R., Goodman S., Hayward R., Ioannidis J.P., Patrick-Lake B., Morton S., Pencina M. (2020). The Predictive Approaches to Treatment effect Heterogeneity (PATH) Statement. Ann. Intern. Med..

[16] Johnston K.L., Thomas E.L., Bell J.D., Frost G.S., Robertson M.D. (2010). Resistant starch improves insulin sensitivity in metabolic syndrome. Diabet. Med..

[17] Bodinham C.L., Smith L., Thomas E.L., Bell J.D., Swann J.R., Costabile A., Russell-Jones D., Umpleby A.M., Robertson M.D. (2014). Efficacy of increased resistant starch consumption in human type 2 diabetes. Endocr. Connect..

[18] Peterson C.M., Beyl R.A., Marlatt K.L., Martin C.K., Aryana K.J., Marco M.L., Martin R.J., Keenan M.J., Ravussin E. (2018). Effect of 12 wk of resistant starch supplementation on cardiometabolic risk factors in adults with prediabetes: A randomized controlled trial. Am. J. Clin. Nutr..

[19] Schioldan A.G., Gregersen S., Hald S., Bjørnshave A., Bohl M., Hartmann B., Holst J.J., Stødkilde-Jørgensen H., Hermansen K. (2018). Effects of a diet rich in arabinoxylan and resistant starch compared with a diet rich in refined carbohydrates on postprandial metabolism and features of the metabolic syndrome. Eur. J. Nutr..

[20] Hald S., Schioldan A.G., Moore M.E., Dige A., Lærke H.N., Agnholt J., Knudsen K.E.B., Hermansen K., Marco M.L., Gregersen S. (2016). Effects of Arabinoxylan and Resistant Starch on Intestinal Microbiota and Short-Chain Fatty Acids in Subjects with Metabolic Syndrome: A Randomised Crossover Study. PLoS ONE.

[21] Bomhof M.R., Parnell J.A., Ramay H.R., Crotty P., Rioux K.P., Probert C.S., Jayakumar S., Raman M., Reimer R.A. (2019). Histological improvement of non-alcoholic steatohepatitis with a prebiotic: A pilot clinical trial. Eur. J. Nutr..

[22] Reshef N., Gophna U., Reshef L., Konikoff F., Gabay G., Zornitzki T., Knobler H., Maor Y. (2024). Prebiotic Treatment in Patients with Nonalcoholic Fatty Liver Disease (NAFLD)—A Randomized Pilot Trial. Nutrients.

[23] Mayengbam S., Raman M., Parnell J.A., Eksteen B., Lambert J.E., Eller L.K., Nicolucci A.C., Aktary M.L., Reimer R.A. (2025). Effects of combined prebiotic fiber supplementation and weight loss counseling in adults with metabolic dysfunction-associated steatotic liver disease: A randomized controlled trial. Eur. J. Nutr..

[24] Chambers E.S., Byrne C.S., Rd A.R., Morrison D.J., Preston T., Tedford C., Bell J.D., Thomas L., Akbar A.N., Riddell N.E. (2019). The effects of dietary supplementation with inulin and inulin-propionate ester on hepatic steatosis in adults with non-alcoholic fatty liver disease. Diabetes Obes. Metab..

[25] Hughes R.L., Horn W.H., Finnegan P., Newman J.W., Marco M.L., Keim N.L., Kable M.E. (2021). Resistant Starch Type 2 from Wheat Reduces Postprandial Glycemic Response with Concurrent Alterations in Gut Microbiota Composition. Nutrients.

[26] Cao S., Shaw E.L., Quarles W.R., Sasaki G.Y., Dey P., Hodges J.K., Pokala A., Zeng M., Bruno R.S. (2022). Daily Inclusion of Resistant Starch-Containing Potatoes in a Dietary Guidelines for Americans Dietary Pattern Does Not Adversely Affect Cardiometabolic Risk or Intestinal Permeability in Adults with Metabolic Syndrome: A Randomized Controlled Trial. Nutrients.

[27] Gondalia S.V., Wymond B., Benassi-Evans B., Berbezy P., Bird A.R., Belobrajdic D.P. (2022). Substitution of Refined Conventional Wheat Flour with Wheat High in Resistant Starch Modulates the Intestinal Microbiota and Fecal Metabolites in Healthy Adults: A Randomized, Controlled Trial. J. Nutr..

[28] Iwata R., Otomo Y., Nishitsuji Y., Node J., Toyota K., Ebihara S., Kikuchi Y. (2026). In Vivo and In Vitro Effects of Fermentable Dietary Fiber from High-Amylose Wheat Containing Resistant Starch on the Intestinal Environment: A Randomized, Double-Blind, Placebo-Controlled, Human Trial. Microorganisms.

[29] Bodinham C.L., Smith L., Wright J., Frost G.S., Robertson M.D. (2012). Dietary Fibre Improves First-phase Insulin Secretion in Overweight Individuals. PLoS ONE.

[30] Li H., Zhang L., Li J., Wu Q., Qian L., He J., Ni Y., Kovatcheva-Datchary P., Yuan R., Liu S. (2024). Resistant starch intake facilitates weight loss in humans by reshaping the gut microbiota. Nat. Metab..

[31] Malcomson F.C., Louca P., Nelson A., Willis N.D., McCallum I., Xie L., Ouwehand A.C., Stowell J.D., Preston T., Morrison D.J. (2024). Effects of non-digestible carbohydrates on gut microbiota and microbial metabolites: A randomised, controlled dietary intervention in healthy individuals. Br. J. Nutr..

[32] Eshghi F., Bakhshimoghaddam F., Rasmi Y., Alizadeh M. (2019). Effects of Resistant Starch Supplementation on Glucose Metabolism, Lipid Profile, Lipid Peroxidation Marker, and Oxidative Stress in Overweight and Obese Adults: Randomized, Double-Blind, Crossover Trial. Clin. Nutr. Res..

[33] Kim K.-N., Kang M.-S., Joo N.-S., Lee H.-R., Jung S., Ju S., Lee Y.-J., Lee K., Jung S., Im J. (2025). Effects of Resistant Starch on Metabolic Markers and Gut Microbiota in Women with Metabolic Syndrome Risk Factors: A Randomized, Double-Blind, Pilot Study. Nutrients.

[34] Lancaster S.M., Lee-McMullen B., Abbott C.W., Quijada J.V., Hornburg D., Park H., Perelman D., Peterson D.J., Tang M., Robinson A. (2022). Global, distinctive, and personal changes in molecular and microbial profiles by specific fibers in humans. Cell Host Microbe.

[35] Lazo M., Solga S.F., Horska A., Bonekamp S., Diehl A.M., Brancati F.L., Wagenknecht L.E., Pi-Sunyer F.X., Kahn S.E., Clark J.M. (2010). Effect of a 12-Month Intensive Lifestyle Intervention on Hepatic Steatosis in Adults With Type 2 Diabetes. Diabetes Care.

[36] Ryan M.C., Itsiopoulos C., Thodis T., Ward G., Trost N., Hofferberth S., O’dEa K., Desmond P.V., Johnson N.A., Wilson A.M. (2013). The Mediterranean diet improves hepatic steatosis and insulin sensitivity in individuals with non-alcoholic fatty liver disease. J. Hepatol..

[37] Martínez I., Kim J., Duffy P.R., Schlegel V.L., Walter J. (2010). Resistant Starches Types 2 and 4 Have Differential Effects on the Composition of the Fecal Microbiota in Human Subjects. PLoS ONE.

[38] Devarakonda S.L.S., Superdock D.K., Ren J., Johnson L.M., Loinard-González A.P., Poole A.C. (2024). Gut microbial features and dietary fiber intake predict gut microbiota response to resistant starch supplementation. Gut Microbes.

[39] Piperni E., Blanco-Míguez A., Mengoni C., Piccinno G., Punčochář M., Ren J., Segata N., Asnicar F., Poole A.C. (2026). Resistant starch types 2 and 4 induce distinct and reversible changes in the human gut microbiome. Microbiol. Spectr..

[40] Deehan E.C., Yang C., Perez-Muñoz M.E., Nguyen N.K., Cheng C.C., Triador L., Zhang Z., Bakal J.A., Walter J. (2020). Precision Microbiome Modulation with Discrete Dietary Fiber Structures Directs Short-Chain Fatty Acid Production. Cell Host Microbe.

[41] Teichmann J., Cockburn D.W. (2021). In vitro Fermentation Reveals Changes in Butyrate Production Dependent on Resistant Starch Source and Microbiome Composition. Front. Microbiol..

[42] Marcia A.D.R., Yao T., Chen M.-H., Oles R.E., Lindemann S.R. (2021). Fine Carbohydrate Structure of Dietary Resistant Glucans Governs the Structure and Function of Human Gut Microbiota. Nutrients.

[43] Li S., Meng Y., Wang C., Suonan Z., Zhang X., Wu T., Dai Z., Zhang Y., Sharafeldin S., Zhang Y. (2023). Effect of structural characteristics of resistant starch prepared by various methods on microbial community and fermentative products. Int. J. Biol. Macromol..

[44] Wang C., Chao C., Sun R., Yu J., Yang Y., Copeland L., Wang S. (2025). Increased Crystallite Stability Enhances Gut Microbial Fermentability of Type 5 Resistant Starch. J. Agric. Food Chem..

[45] Ze X., Duncan S.H., Louis P., Flint H.J. (2012). Ruminococcus bromii is a keystone species for the degradation of resistant starch in the human colon. ISME J..

[46] Abell G.C.J., Cooke C.M., Bennett C.N., Conlon M.A., McOrist A.L. (2008). Phylotypes related to Ruminococcus bromii are abundant in the large bowel of humans and increase in response to a diet high in resistant starch. FEMS Microbiol. Ecol..

[47] Walker A.W., Ince J., Duncan S.H., Webster L.M., Holtrop G., Ze X., Brown D., Stares M.D., Scott P., Bergerat A. (2010). Dominant and diet-responsive groups of bacteria within the human colonic microbiota. ISME J..

[48] Wimmer B.H., Moraïs S., Amit I., Tovar-Herrera O., Tatli M., Trautwein-Schult A., Pfister B., Zalk R., Tödtli P., Simoni S. (2025). Spatial constraints drive amylosome-mediated resistant starch degradation by Ruminococcus bromii in the human colon. Nat. Commun..

[49] Li L., Ryan J., Ning Z., Zhang X., Mayne J., Lavallée-Adam M., Stintzi A., Figeys D. (2020). A functional ecological network based on metaproteomics responses of individual gut microbiomes to resistant starches. Comput. Struct. Biotechnol. J..

[50] Dobranowski P., Duan H., Butcher J., Mayne J., Figeys D., Mack D.R., Stintzi A. (2026). Individual variability shapes ex vivo responses to resistant starch in inflammatory bowel disease derived microbiomes. npj Biofilms Microbiomes.

[51] Swanson K.S., Gibson G.R., Hutkins R., Reimer R.A., Reid G., Verbeke K., Scott K.P., Holscher H.D., Azad M.B., Delzenne N.M. (2020). The International Scientific Association for Probiotics and Prebiotics (ISAPP) consensus statement on the definition and scope of synbiotics. Nat. Rev. Gastroenterol. Hepatol..

[52] Kovatcheva-Datchary P., Nilsson A., Akrami R., Lee Y.S., De Vadder F., Arora T., Hallen A., Martens E., Björck I., Bäckhed F. (2015). Dietary Fiber-Induced Improvement in Glucose Metabolism Is Associated with Increased Abundance of Prevotella. Cell Metab..

[53] Christensen L., Vuholm S., Roager H.M., Nielsen D.S., Krych L., Kristensen M., Astrup A., Hjorth M.F. (2019). Prevotella Abundance Predicts Weight Loss Success in Healthy, Overweight Adults Consuming a Whole-Grain Diet Ad Libitum: A Post Hoc Analysis of a 6-Wk Randomized Controlled Trial. J. Nutr..

[54] Blanco-Míguez A., Gálvez E.J.C., Pasolli E., De Filippis F., Amend L., Huang K.D., Manghi P., Lesker T.-R., Riedel T., Cova L. (2023). Extension of the Segatella copri complex to 13 species with distinct large extrachromosomal elements and associations with host conditions. Cell Host Microbe.

[55] Vandeputte D., Kathagen G., D’hoe K., Vieira-Silva S., Valles-Colomer M., Sabino J., Wang J., Tito R.Y., De Commer L., Darzi Y. (2017). Quantitative microbiome profiling links gut community variation to microbial load. Nature.

[56] Alfa M.J., Strang D., Tappia P.S., Graham M., Van Domselaar G., Forbes J.D., Laminman V., Olson N., DeGagne P., Bray D. (2018). A randomized trial to determine the impact of a digestion resistant starch composition on the gut microbiome in older and mid-age adults. Clin. Nutr..

[57] Bartsch M., Vital M., Woltemate S., Bouwman F.G., Berkemeyer S.B., Hahn A., Müller M. (2025). Microbiota-Dependent Fiber Responses: A Proof-of-Concept Study on Short-Chain Fatty Acid Production in Prevotella- and Bacteroides-Dominated Healthy Individuals. J. Nutr..

[58] Sardá F.A.H., Giuntini E.B., Oliveira A., Souza G.S., Prado S.B.R., Taddei C.R., Tadini C.C., Bittinger K., Bushman F.D., Menezes E.W. (2025). Baseline intestinal microbiota composition influences response to a real-world dietary fiber intervention. npj Biofilms Microbiomes.

[59] Dong B., Ye M., Xu Z.Z., Huang X., Hu Y. (2026). Resistant starch selectively depletes a putative pathobiont-enriched gut microbial module: Evidence from multiple dietary fiber intervention cohorts. Front. Nutr..

[60] Vandeputte D., Falony G., Vieira-Silva S., Tito R.Y., Joossens M., Raes J. (2016). Stool consistency is strongly associated with gut microbiota richness and composition, enterotypes and bacterial growth rates. Gut.

[61] Procházková N., Laursen M.F., La Barbera G., Tsekitsidi E., Jørgensen M.S., Rasmussen M.A., Raes J., Licht T.R., Dragsted L.O., Roager H.M. (2024). Gut physiology and environment explain variations in human gut microbiome composition and metabolism. Nat. Microbiol..

[62] Zhang L., Ouyang Y., Li H., Shen L., Ni Y., Fang Q., Wu G., Qian L., Xiao Y., Zhang J. (2019). Metabolic phenotypes and the gut microbiota in response to dietary resistant starch type 2 in normal-weight subjects: A randomized crossover trial. Sci. Rep..

[63] Fong J.V.N., Miketinas D., Moore L.W., Nguyen D.T., Graviss E.A., Ajami N., Patterson M.A. (2022). Precision Nutrition Model Predicts Glucose Control of Overweight Females Following the Consumption of Potatoes High in Resistant Starch. Nutrients.

[64] Boets E., Gomand S.V., Deroover L., Preston T., Vermeulen K., De Preter V., Hamer H.M., Van den Mooter G., De Vuyst L., Courtin C.M. (2017). Systemic availability and metabolism of colonic-derived short-chain fatty acids in healthy subjects: A stable isotope study. J. Physiol..

[65] Tolhurst G., Heffron H., Lam Y.S., Parker H.E., Habib A.M., Diakogiannaki E., Cameron J., Grosse J., Reimann F., Gribble F.M. (2012). Short-Chain Fatty Acids Stimulate Glucagon-Like Peptide-1 Secretion via the G-Protein-Coupled Receptor FFAR2. Diabetes.

[66] Venkataraman A., Sieber J.R., Schmidt A.W., Waldron C., Theis K.R., Schmidt T.M. (2016). Variable responses of human microbiomes to dietary supplementation with resistant starch. Microbiome.

[67] Zhang R., Mu H., Li Z., Zeng J., Zhou Q., Li H., Wang S., Li X., Zhao X., Sun L. (2022). Oral administration of branched-chain amino acids ameliorates high-fat diet-induced metabolic-associated fatty liver disease via gut microbiota-associated mechanisms. Front. Microbiol..

[68] Iwao M., Gotoh K., Arakawa M., Endo M., Honda K., Seike M., Murakami K., Shibata H. (2020). Supplementation of branched-chain amino acids decreases fat accumulation in the liver through intestinal microbiota-mediated production of acetic acid. Sci. Rep..

[69] Serin S., Sayar S. (2023). In vitro digestibility, glycaemic index and bile acid–binding capacity of foods containing different types of resistant starch in comparison with the commercial resistant starches. Qual. Assur. Saf. Crop. Foods.

[70] Fan J., Le Gall G., Malcomson F.C., Louca P., Beck L., Nelson A., Willis N.D., McCallum I., Xie L., Ouwehand A.C. (2026). Distinct effects of supplementation with resistant starch and polydextrose on plasma and faecal bile acid profile and associations with gut microbiota: A randomised, controlled intervention in healthy participants. Eur. J. Nutr..

[71] Upadhyaya B., McCormack L., Fardin-Kia A.R., Juenemann R., Nichenametla S., Clapper J., Specker B., Dey M. (2016). Impact of dietary resistant starch type 4 on human gut microbiota and immunometabolic functions. Sci. Rep..

[72] Osawa R., Inoue J., Fukuda I., Shirai Y., Kodama Y. (2026). Function-based personalization of dietary fiber selection enhances gut microbial fermentation: A proof-of-concept crossover trial. Front. Nutr..

[73] Zeevi D., Korem T., Zmora N., Israeli D., Rothschild D., Weinberger A., Ben-Yacov O., Lador D., Avnit-Sagi T., Lotan-Pompan M. (2015). Personalized Nutrition by Prediction of Glycemic Responses. Cell.

[74] Mendes-Soares H., Raveh-Sadka T., Azulay S., Ben-Shlomo Y., Cohen Y., Ofek T., Stevens J., Bachrach D., Kashyap P., Segal L. (2019). Model of personalized postprandial glycemic response to food developed for an Israeli cohort predicts responses in Midwestern American individuals. Am. J. Clin. Nutr..

[75] Bermingham K.M., Linenberg I., Polidori L., Asnicar F., Arrè A., Wolf J., Badri F., Bernard H., Capdevila J., Bulsiewicz W.J. (2024). Effects of a personalized nutrition program on cardiometabolic health: A randomized controlled trial. Nat. Med..

[76] Popp C.J., Hu L., Kharmats A.Y., Curran M., Berube L., Wang C., Pompeii M.L., Illiano P., St-Jules D.E., Mottern M. (2022). Effect of a Personalized Diet to Reduce Postprandial Glycemic Response vs a Low-fat Diet on Weight Loss in Adults With Abnormal Glucose Metabolism and Obesity: A Randomized Clinical Trial. JAMA Netw. Open.

[77] Song D., Feng G., Ma Y., Shi Y., Qian C., Wang C., Xu J., Li Y., Wang X., Fan N. (2025). Gut microbiome predicts personalized responses to dietary fiber in prediabetes: A randomized, open-label trial. Nat. Commun..

[78] Wastyk H.C., Fragiadakis G.K., Perelman D., Dahan D., Merrill B.D., Yu F.B., Topf M., Gonzalez C.G., Van Treuren W., Han S. (2021). Gut-microbiota-targeted diets modulate human immune status. Cell.

[79] Collins G.S., Moons K.G.M., Dhiman P., Riley R.D., Beam A.L., Van Calster B., Ghassemi M., Liu X., Reitsma J.B., van Smeden M. (2024). TRIPOD+AI statement: Updated guidance for reporting clinical prediction models that use regression or machine learning methods. BMJ.

[80] Moons K.G.M., Damen J.A.A., Kaul T., Hooft L., Navarro C.A., Dhiman P., Beam A.L., Van Calster B., Celi L.A., Denaxas S. (2025). PROBAST+AI: An updated quality, risk of bias, and applicability assessment tool for prediction models using regression or artificial intelligence methods. BMJ.

[81] Van Calster B., McLernon D.J., van Smeden M., Wynants L., Steyerberg E.W. (2019). Topic Group “Evaluating diagnostic tests and prediction models” of the STRATOS initiative. Calibration: The Achilles heel of predictive analytics. BMC Med..

[82] Vickers A.J., Elkin E.B. (2006). Decision Curve Analysis: A Novel Method for Evaluating Prediction Models. Med. Decis. Mak..

